# Pretreatment biomarkers as prognostic predictors of survival in patients with Pancreatic Cancer treated with Gemcitabine-based Therapy and 5-Fluorouracil: Neutrophil-to-lymphocyte ratio *vs* Platelet-to-lymphocyte ratio

**DOI:** 10.7150/ijms.46254

**Published:** 2020-06-06

**Authors:** Yungu Chen, Yuan Liao, Lek Man Lam, Lina He, Yiu Sing Tsang, Ying-San Di, Sheng-Tao Liang, Qing Xia

**Affiliations:** 1 Department of Oncology, State Key Laboratory for Oncogenes and Related Genes, Renji Hospital, School of Medicine, Shanghai Jiaotong University, Shanghai Cancer Institute, Shanghai, 200127, China.; 2 Department of Laboratory Medicine, The Third Affiliated Hospital of Sun Yat-sen University, Guangzhou, 510630, China.; 3 Department of Basic Medical Sciences, Shanghai Jiao Tong University School of Medicine, Shanghai, 200025, China.; 4 Department of Oncology, Baoshan Branch Hospital, Renji Hospital, School of Medicine, Shanghai Jiaotong University, Shanghai, 200436, China.

**Keywords:** neutrophil-to-lymphocyte ratio, platelet-to-lymphocyte ratio, pancreatic cancer, prognosis, gemcitabine, 5-fluorouracil

## Abstract

Although elevated neutrophil-to-lymphocyte ratio (NLR) and platelet-to-lymphocyte ratio (PLR) have been reported to be inverse prognostic predictors of survival in patients with pancreatic cancer (PC), the comparison of their prognostic roles in patients with PC undergoing gemcitabine-based chemotherapy and 5-fluorouracil (5-FU) remains unclear. This study was designed and performed to determine the predictive roles of NLR and PLR in patients diagnosed with PC who underwent one of these two regimens. We retrospectively enrolled 95 patients diagnosed with PC undergoing supportive care, gemcitabine-based chemotherapy or 5-FU therapy from January 2015 to October 2018. Univariate and multivariate Cox regression analyses were done to identify clinicopathological predictors of time to treatment failure (TTF) and overall survival (OS), including pretreatment NLR and PLR. The statistical data showed that pretreatment NLR was significantly associated with metastasis. Among all analyzed variables, pretreatment NLR was an independent prognostic predictor of both TTF and OS of patients with PC, with NLR>4.0 predicting worse survival. PLR, however, didn't independently predict TTF or OS. There were no significant difference in the OS of patients undergoing gemcitabine-based regimens and 5-FU regimens when divided into two subgroups: NLR ≤4.0 and >4.0. In conclusion, pretreatment NLR is a promising independent outcome predictor for patients with PC, while NLR might not be a suitable factor in the selection of regimens for patients with PC.

## Introduction

Pancreatic cancer (PC) is one of the most lethal cancers that remains a challenging medical problem for many years. The most common form is pancreatic ductal adenocarcinoma (PDA), the tenth most common solid cancer and the fourth leading cause of death from cancer in the United States 1. The prognosis of PC is very poor, with the 5-year survival rate of only 7% 2. Unfortunately, there are no specific symptoms. Patients at early stage are usually symptom-free, and PC in those who present with unspecific abdominal discomfort, weight loss or more specific jaundice are already in advanced stage. Most patients are diagnosed with metastatic disease and few show a sustained response to chemo- or radiation therapy 3.

Considering the poor prognostic outcome of patients with PC, efforts have been made to explore the predictive factors of this malignancy. It has been recognized that inflammatory responses play decisive roles at different stages of tumor development 4, and several inflammatory biomarkers have been proposed for the evaluation of cancer patients. Recently, pretreatment neutrophil-to-lymphocyte ratio (NLR) has been revealed to be a promising prognostic predictor for pancreatic cancer by several studies, where low NLR stands for better survival in patients with pancreatic cancer 5, 6. NLR is calculated by neutrophil count divided by lymphocyte count, which can be easily obtained by routine blood tests. Previous studies indicated that a high level of NLR might be significantly associated with poorer prognosis of several tumors, including colorectal cancer, lung cancer, gastric cancer, esophageal cancer and so on 7-10. There are also evidences suggesting that platelet-to-lymphocyte (PLR), as another easily accessible biomarker that is calculated by platelet count divided by lymphocyte count, may also be a prognostic predictor of a variety of malignancies such as ovarian cancer 11, breast cancer 12 and non-small-cell lung cancer 13, where low PLR suggests a better outcome.

This study was designed and performed to determine the association between both pretreatment NLR and PLR and survival of patients with PC treated with supportive care, gemcitabine-based chemotherapy or 5-FU therapy.

## Materials and Methods

### Patients and treatment

Searching the database system of electronic medical charts, we collected clinical data of patients with the diagnosis of pancreatic cancer referred to Renji Hospital affiliated to Shanghai Jiao Tong University School of Medicine between January 2015 and October 2018. Patients whose diagnosis was PC were included in the study. Patients without available data or those with infection, regimens with steroids or aspirin, autoimmune disease or other conditions that might possibly confounded neutrophil count, lymphocyte count or platelet were excluded. In this study, patients underwent supportive care, gemcitabine-based therapy or 5-FU therapy. Gemcitabine-based therapy included gemcitabine monotherapy and gemcitabine combination therapy. Treatment was not terminated until tumor recurrence, tumor progression, treatment toxicity or patients' requirement for withdrawal. The doses and regimens were adjusted by corresponding physicians in accordance with adverse events and general conditions of the individual patient.

### Clinical and laboratory data collection

Patients included were either chemotherapy-naïve or chemotherapy-free for at least one month before they were referred to Renji Hospital affiliated to Shanghai Jiao Tong University. Baseline data was collected before treatment start. The history of patients was taken on the first days of hospitalization. Karnofsky performance status (KPS) was evaluated by treating physicians before the commencement of chemotherapy. Tumor location and TNM (tumor, lymph node and metastasis) staging were judged from the results of computed tomography (CT) or positron emission tomography (PET) in accordance with the 7^th^ edition of the *AJCC Cancer Staging Manual*. Biological markers, including carbohydrate antigen 19-9 (CA 19-9), carcinoembryonic antigen (CEA), carbohydrate antigen-125 (CA-125), neutrophil count, lymphocyte count and platelet count were collected within 3 weeks before the first cycle of chemotherapy as the pretreatment data. NLR was defined by the absolute neutrophil count divided by the absolute lymphocyte count. Likewise, PLR was defined by the absolute platelet count divided by the absolute lymphocyte count. All consecutive parameters were categorized for the further analysis as follows: age (≤65 or >65 years), body mass index (BMI) (≤18.5, 18.5-24.0 or >24.0), CA 19-9 (≤1000 or >1000 U/ml), CEA (≤5 or >5 ng/ml), CA-125 (≤38 or >38 U/ml). Cut-off values were set on the basis of previous studies 14, 15.

### Statistical analysis

The χ^2^ test or Fisher's exact test were used to compare baseline patient characteristics that were categorized variables. The association between pretreatment NLR and the time to treatment failure (TTF) as well as overall survival (OS) was evaluated, so was pretreatment PLR. TTF was defined as the time from the date of chemotherapy initiation to the date of termination due to various reasons, including tumor recurrence, tumor progression, treatment toxicity or patients' requirement for withdrawal. If a patient had not reached the endpoint caused by any of these reasons, TTF was censored at the time of the last follow-up. Recurrence and progression were determined using CT or PET. OS was calculated from the date of chemotherapy initiation to the date of death for any reason, or censored at the date of the last follow-up if the endpoint event was not observed. Univariate and multivariate Cox regression analyses were done to identify clinicopathological predictors of TTF and OS, including age, gender, BMI, KPS, personal history, diabetes at diagnosis, tumor location, TNM stage, CA 19-9, CEA, CA-125 and pretreatment NLR and PLR. The differences of TTF and OS were compared utilizing the Kaplan-Meier method with log-rank tests for survival plot depiction and Cox-regression analysis for the evaluation of hazard ratio (HR) and its 95% confidence interval (95% CI).

In order to set the cut-off points of both NLR and PLR, Receiver Operating Curves (ROC) were depicted. The classification variable was long vs short-term survival (>6 vs ≤6 months). For one thing, the choice of the 6 months as the division point was due to the convenience of the study, since a relevant proportion of patients (47.4%) were classified as short-term survivors and only 4 patients had a follow-up less than 6 months. For another, the majority of patients included in this study were in stage IV, whose median survival was only 4-6 months 16, so the choice of the 6 months was feasible. The effects of potential prognostic predictors were tested by Cox regression. Only variables with a statistical significance in univariate analysis were investigated in multivariate analysis.

The statistical significance of all tests was two-sided, *p*<0.05. Analyses and calculation were done by IBM SPSS Statistics 24.0.

### Regulatory consideration

This study is approved by the Ethic Committee of Renji Hospital affiliated to Shanghai Jiao Tong University as stipulated by the Declaration of Helsinki.

## Results

A total of 103 patients diagnosed with pancreatic cancer were referred to Renji Hospital affiliated to Shanghai Jiao Tong University School of Medicine between January 2015 and October 2018. All patients were histologically confirmed to have PC by biopsy, from which 4 patients with unavailable data and 4 patients who had infections, steroid regimens or autoimmune disease at the time of baseline data collection were excluded. Finnally, 95 patients were enrolled in the study.

The demographics and characteristics of all patients were shown in Table 1. Among the 95 patients in the study, 56 were male and 39 were female. The median age at baseline was 62 years (range, 42-83). BMI ranged from 14.4 to 28.1 kg/m^2^, with the median of 20.8 kg/m^2^ and 42.1% patients had KPS≤80. As for the investigation of risk factors of PC, 32.6% of patients had a history of smoking while 16.8% had a history of alcohol intake. Moreover, 13.7% of patients had a family history of cancer and 32.6% patients had diabetes at the time of PC diagnosis. Histologically, 87.4 % patients were confirmed to have PC while others had neuroendocrine tumor and mucinous carcinoma. Tumor in 42.1% patients located in the head of pancreas and distantly metastasized in 72.6% patients. Routine biochemical tests performed before the commencement of treatment were collected as baseline data, some of which were long recognized prognostic predictors. In terms of therapy, 12.6% patients underwent supportive care, whereas 53.7% patients were treated by gemcitabine-based therapy and 33.7% patients by 5-FU. The median neutrophil count was 3.60*10^9^/L, with the range of 1.44 - 15.75*10^9^/L. The median lymphocyte count was 1.42*10^9^/L, ranging from 0.50*10^9^ to 4.50*10^9^/L. Median NLR was 2.6 (range, 0.5-18.0) with 27 (28.4%) patients having NLR>4.0 and that of PLR was 148 (range, 51-785) with 39 (41.1%) patients having PLR>169. Among all 95 patients, 39 (41.1%) presented with the biomarker CA 19-9>1000 U/ml, 47 (49.5%) with CEA>5 ng/ml and 49 (51.6%) with CA-125>38 U/ml.

**Figure 1:**
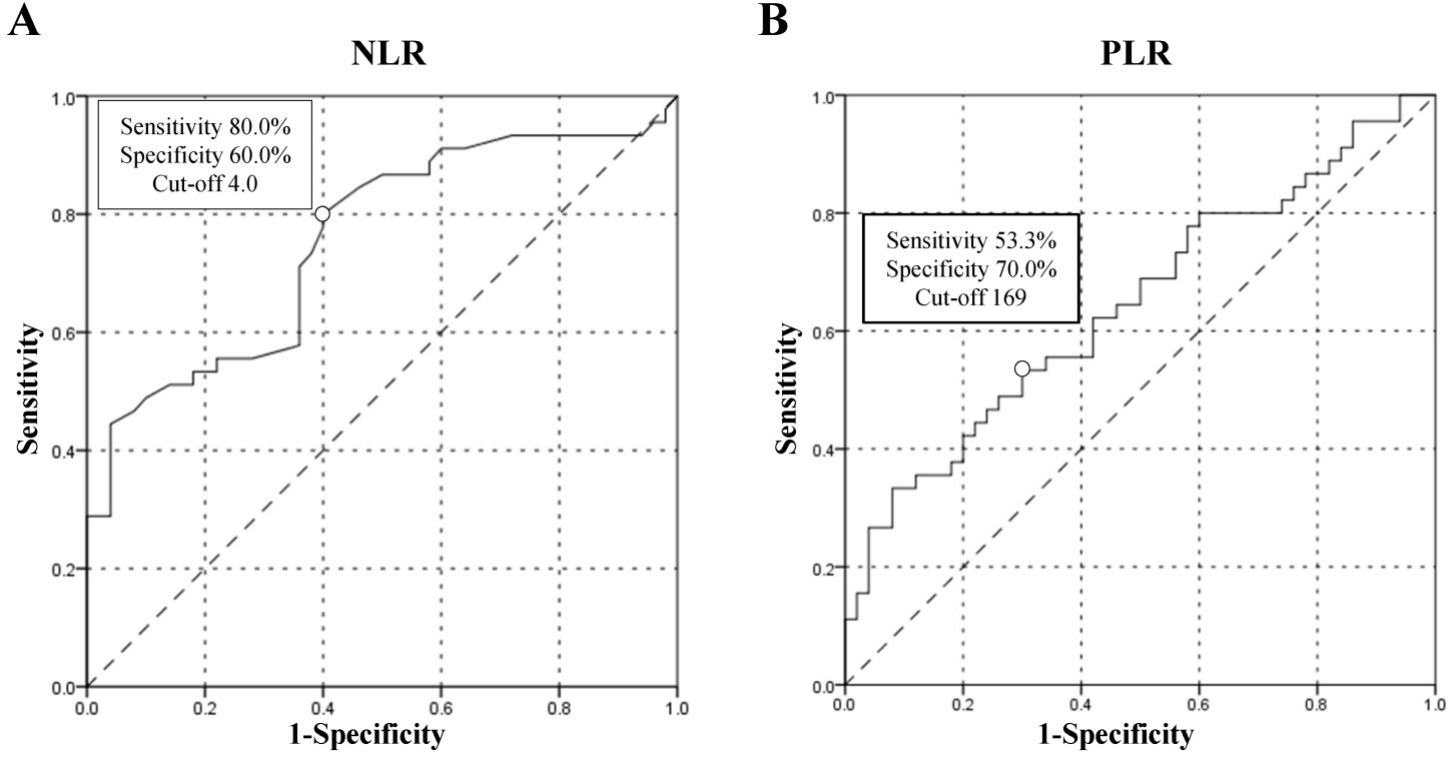
Receiver Operating Curve (ROC) analysis constructed to find the best cut-off point of neutrophil-to-lymphocyte ratio (NLR) and platelet-to-lymphocyte ratio (PLR). (A) ROC analysis of NLR. (B) ROC analysis of PLR.

**Figure 2:**
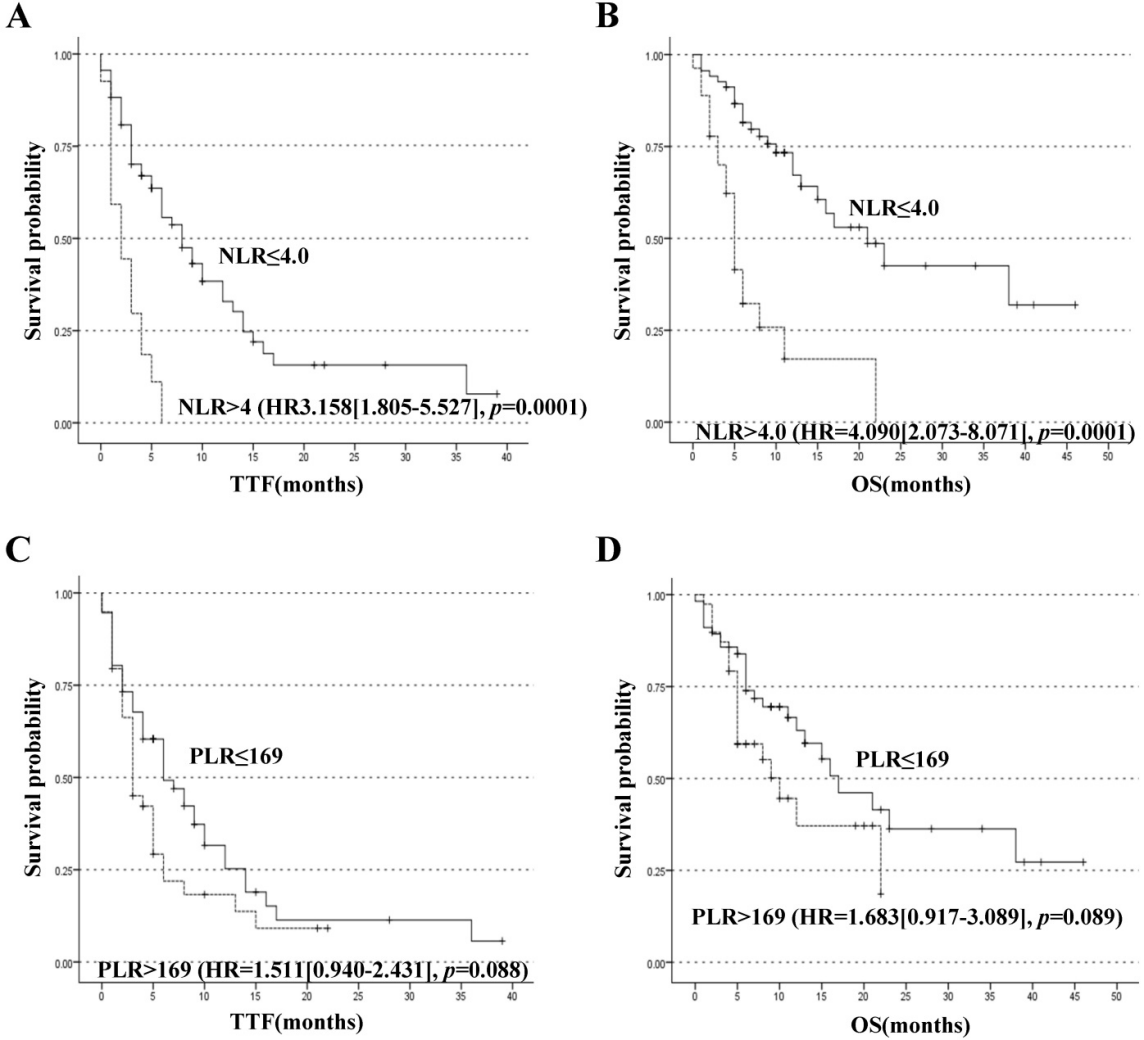
Kaplan-Meier curves stratified by the pretreatment levels of NLR and PLR. (A) Time to treatment failure (TTF) and (B) Overall survival (OS) of patients classified by NLR≤4.0 or >4.0. (C)TTF and (D) OS of patients classified by PLR≤169 or > 169.

Until October 2018, 72 patients reached TTF endpoint and 45 patients reached OS endpoint. For better utility of NLR as a predictor for the prognosis of patients with PC, a ROC curve was constructed to find the cut-off point of NLR. The area under curve (AUC) was 0.754. A fixed cut-off value of 4.0 was taken for the analysis, yielding a sensitivity of 80.0% and a specificity of 60.0% (Figure 1A). With NLR=4.0 as the cut-off point, it was confirmed that pretreatment NLR was significantly associated with TTF, with median TTF of 8.0 months and 2.0 months for patients with NLR≤4.0 and >4.0 respectively, HR=3.158 (95% CI, 1.805-5.527), *p*=0.0001 (Figure 2A). NLR was also significantly associated with OS. The median OS of patients with NLR≤4.0 was 21.0 months and that of patients with NLR>4.0 was 5.0 months, HR=4.090 (HR=2.073-8.071), *p*=0.0001 (Figure 2B). A ROC curve was also constructed to find the appropriate cut-off point of PLR. The AUC was 0.645. PLR=169 was found to be the best cut-off point in this study, with a sensitivity of 53.3% and a specificity of 70.0% (Figure 1B). Median TTF of PLR≤169 and >169 was 6.0 months and 3.0 months respectively, HR=1.511 (95% CI, 0.940-2.431), *p*=0.088 (Figure 2C). And median OS of PLR≤169 and >169 was 17.0 months and 10.0 months respectively, HR=1.683 (95% CI, 0.917-3.089), *p*=0.089 (Figure 2D). The associations between NLR subgroups and biomarker CA 19-9 (*p*=0.672), CEA (*p*=0.455) and CA-125 (*p*=0.625) were found not of significance (Table 1).

It was uncovered that NLR was significantly associated with M stage, where NLR>4.0 suggested a more likely metastatic scenario (*p*=0.001). And in those patients with localized tumors at the time of diagnosis, their NLR was almost all below 4.0 (25/26). Such an association might partially explain the association between NLR and prognosis for patients with metastatic PC who undoubtedly tend to have a worse prognosis. Nevertheless, PLR was not found to be significantly associated with tumor metastasis (*p*=0.211).

To evaluate whether NLR and PLR were independent predictors of prognosis, univariate and multivariate Cox-regression analyses for both TTF and OS were performed (Table 2). In the assessment of potential prognostic variables of TTF, N stage, M stage, CA 19-9, CA-125, CEA, pretreatment NLR were found to be significantly associated with TTF at univariate Cox-regression analysis. At multivariate analysis, only NLR had statistically significant association with TTF, the HR of NLR being 3.158 (95% CI, 1.805-5.527), *p*=0.0001, although M stage exhibited a marginal significance (*p*=0.051). At univariate analysis of OS, it was found that N stage, M stage, CA 19-9, CA-125, CEA, pretreatment NLR were significantly associated with OS. And NLR was proved to be an independent prognostic factor of OS, HR being 4.090 (95% CI 2.073-8.071). *p*=0.0001. Meanwhile N stage also presented a statistical significance (*p*=0.012). In univariable analysis, pretreatment PLR didn't show any significant association with TTF (HR=1.511[0.940-2.431], *p*=0.088, Figure 2C) or OS (HR=1.683[0.917-3.089], *p*=0.089, Figure 2D).

**Figure 3:**
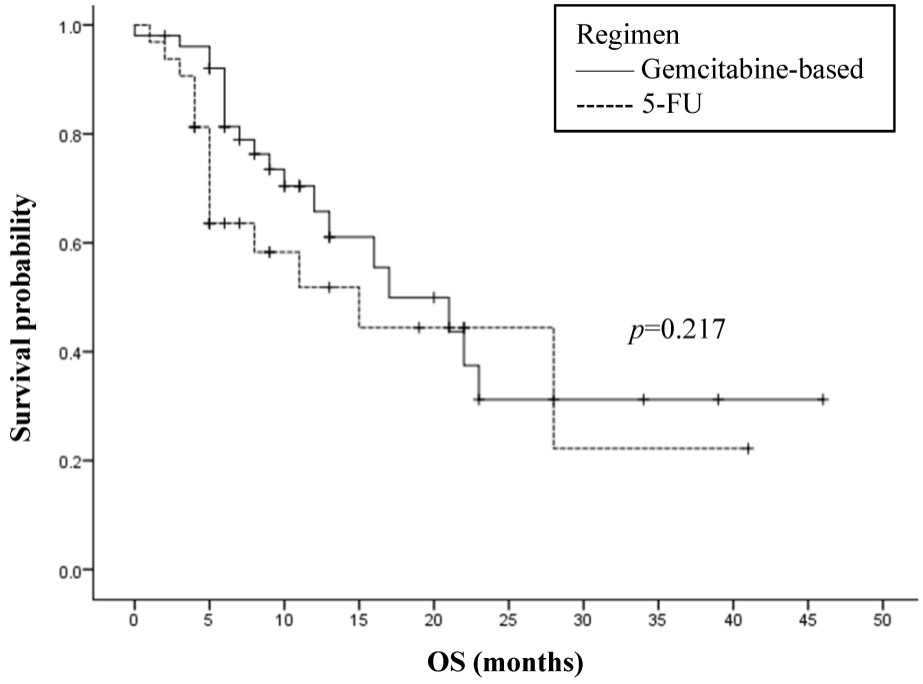
Overall survival (OS) and first-line chemotherapy regimen in 83 analyzed patients. 5-FU: 5-fluorouracil.

Among the 83 patients selected for the study that underwent chemotherapy, the regimens of 51 patients were gemcitabine-based and that of the others received 5-FU containing regimens. Gemcitabine-based therapy displayed a minor superior OS when compared to 5-FU, with median OS of 17.0 months and 15.0 months respectively (HR=1.525[0.776-2.995]). However, this was not of statistical significance (*p*=0.217, Figure 3). To evaluate the influence of NLR on the type of chemotherapy, the association between chemotherapy and OS was analyzed by separating patients into low and high pretreatment NLR. No significant interaction was found, with no difference between gemcitabine-based and 5-FU in the low NLR subgroup (*p*=0.310) and the high NLR subgroup (*p*=0.409).

## Discussion

NLR has been confirmed to be a prognostic predictor in various cancers that significantly correlates with response rates, therapeutic effects and survival rates 7-10. For patients with pancreatic cancer, high pretreatment NLR is found to be an unfavorable predictor of OS and TTF, and some studies pointed out that patients with high pretreatment NLR would tend to have a longer OS or PFS, if their NLR was lowered after the initiation of chemotherapy 17, 18. In the present study, we demonstrated that pretreatment NLR independently predicted the prognosis of patients with advanced or localized PC, even after adjusting for potential confounding factors. But PLR failed to independently predict the prognostic outcome in terms of both TTF and OS.

A number of studies have suggested the inverse association between both NLR and PLR and prognosis of patients with PC 6, 19. Although it has long been established that NLR and PLR both play roles in predicting the prognosis of various malignancies, the question which one is more predictive and suggestive is still debatable. The comparison between NLR and PLR was done to analyze which factor does better in predicting the prognosis of patients with PC.

A dynamic and mutualistic interaction between tumour cells and the surrounding stromal cells promotes the initiation, progression, metastasis and chemoresistance of solid tumours 20. As important components of stromal cells, neutrophils are enriched in many types of cancers and high levels of neutrophils are closely associated with disease progression and poor clinical outcome 21. Neutrophilia is a common phenomenon in PC. Multiple mechanisms have been hypothesized or demonstrated. It was proposed that neutrophil might aid metastasis of pancreatic cancer because it is able to mediate epithelial-mesenchymal transition (EMT) of cancer cells by secreting elastase 22. Neutrophil can secrete vascular endothelial growth factor (VEGF) and matrix metalloproteinase 9 (MMP-9), which promotes angiogenesis thus facilitating the growth and metastases of PC 23. Another study found that neutrophils can also promote tumor growth through converting senescent cancer cells into proliferating cancer cells via IL-1 receptor antagonist 24. Besides, neutrophil may help with the creation of immunosuppressive microenvironment of pancreatic cancer by suppressing CD8^+^T cells 25. Pancreatic cancer is also associated with declined lymphocyte count, which may impair immune surveillance and defense. The proliferation of Lymphocytes is suppressed by various immunosuppressive cytokines during tumorigenesis, principally including transforming growth factor β (TGF-β) and interleukin 10 (IL-10) 26. The association between thrombocytosis and PC is still not well understood. Platelets can secrete tumor growth factors, such as VEGF, TGF-β, platelet-derived growth factor (PDGF), and insulin-like growth factor-1 (IGF1), which play critical roles in cancer angiogenesis and metastasis 27.

In this study, NLR did provide independent prognostic information of survival in terms of both TTF and OS, whereas PLR was not significantly associated with TTF or OS. Several studies have compared the predictive value of NLR and PLR in terms of survival, response rates to treatment and recurrence after resection. NLR was recognized as a promising prognostic predictor whereas the association of PLR with patient outcome seems to be controversial 28. Hasegawa et al. reported that pretreatment NLR might be a useful predictive marker for the pathological response to preoperative therapy in pancreatic cancer patients, which pretreatment PLR failed to predict 29. Martin et al. found both NLR and PLR were predictive of overall survival of patients with advanced PC, despite a more powerful predictive value of NLR (HR=1.81, *p*=0.0007) than PLR (HR=1.64, *p*=0.007) 30. Stotz et al. reported that NLR added independent prognostic information to other well established prognostic factors in patients with pancreatic cancer, regardless of the undergoing therapeutic modality (HR=2.532,* p*<0.001), but PLR did not show any predictive value as for overall survival in patients with both inoperative (*p*=0.612) and operative pancreatic cancer (*p*=0.458) 31. Yang et al. performed a meta-analysis and found that high peripheral blood PLR suggested a poor prognosis for patients with pancreatic cancer 32. Kishi et al. analyzed 65 patients with pancreatic cancer and drew the conclusion that PLR was not associated with the prognosis of these patients 33. As far as this study is concerned, PLR failed to show the same significant association as NLR did. NLR might possess a better predictive value than PLR does in patients with PC.

Early administration of chemotherapy is favoured for patients with advanced PC and the standard of chemotherapy has changed in the last few years in the treatment of PC 34. 5-FU regimens were in common use prior to the adoption of gemcitabine as the chemotherapy for patients with PC. Nonetheless, according to several phase II and randomized controlled trials, both bolus and infusion leucovorin modulated 5-FU-containing regimens are associated with low response rates and survival inferior to that seen with gemcitabine 35, 36. Still, 5-FU remains an alternative for gemcitabine-intolerant patients. In this study, when patients were subgrouped by the criterion of NLR≤4.0 and >4.0, the OS did not significantly vary between gemcitabine-based regimens and 5-FU regimens. NLR might not be a suitable factor in the selection of regimens for patients with PC.

We recognize several limitations in our study. Firstly, the research was retrospective with a relatively small sample size. Besides, the analysis of NLR and PLR for predictive values of different chemotherapeutic modalities was achieved by dividing the population into smaller chemotherapy subgroups. Moreover, the major drawback is that treatment assignment was not randomized. Finally, blood samples were not derived during follow-up, making it infeasible to analyze whether further change of NLR and PLR would predict prognosis more accurately.

In conclusion, pretreatment NLR is a promising independent outcome predictor for patients with pancreatic ductal adenocarcinoma. The predictive value of PLR might not be as good as NLR. NLR could be used to predict time to treatment failure and overall survival for these patients.

## Figures and Tables

**Table 1:** Characteristics of the 95 patients in study

Characteristics	Category (n=95)	Pretreatment NLR ≤4.0 (n=68)	Pretreatment NLR >4.0 (n=27)	*p* value
**Gender**				
Male	56(58.9)	39(57.4)	17(63.0)	
Female	39(41.1)	29(42.6)	10(37.0)	0.586
**Age**				
≤65 years	66(69.5)	46(67.6)	20(74.1)	
>65 years	29(30.5)	22(32.4)	7(25.9)	0.540
**BMI**				
18.5-24.0	66(69.5)	48(70.6)	18(66.7)	
≤18.5	12(12.6)	9(13.2)	3(11.1)	
>24.0	17(17.9)	11(16.2)	6(22.2)	0.776
**KPS**				
≤80	40(42.1)	26(38.2)	14(51.9)	
>80	55(57.9)	42(61.8)	13(48.1)	0.225
**History of Smoking**				
Yes	31(32.6)	19(27.9)	12(44.4)	
No	64(67.4)	49(72.1)	15(55.6)	0.122
**History of Alcohol Intake**				
Yes	16(16.8)	8(11.8)	8(29.6)	
No	79(83.2)	60(88.2)	19(70.4)	0.036
**Family History of Cancer**				
Yes	13(13.7)	9(13.2)	4(14.8)	
No	82(86.3)	59(86.8)	23(85.2)	0.840
**Diabetes at Diagnosis**				
Yes	31(32.6)	23(33.8)	8(29.6)	
No	64(67.4)	45(66.2)	19(70.4)	0.694
**Histology**				
PDA	83(87.4)	61(89.7)	22(81.5)	
Others	12(12.6)	7(10.3)	5(18.5)	0.276
**T Stage**				
T1	8(8.4)	7(10.3)	1(3.7)	
T2	31(32.6)	20(29.4)	11(40.7)	
T3	28(29.5)	20(29.4)	8(29.6)	
T4	28(29.5)	21(30.9)	7(25.9)	0.590
**N Stage**				
N0	25(26.3)	23(33.8)	2(7.4)	
N1	70(73.7)	45(66.2)	25(92.6)	0.008
**M Stage**				
M0	26(27.4)	25(36.8)	1(3.7)	
M1	69(72.6)	43(63.2)	26(96.3)	0.001
**Metastatic Site(s)**				
0	26(27.4)	25(36.8)	1(3.7)	
1	37(38.9)	26(38.2)	11(40.7)	
More than 1	32(33.7)	17(25.0)	15(55.6)	0.001
**Tumor Location**				
Head	40(42.1)	29(42.6)	11(40.7)	
Others	55(57.9)	39(57.4)	16(59.3)	0.865
**CA 19-9**				
≤1000 U/ml	56(58.9)	41(60.3)	15(55.6)	
>1000 U/ml	39(41.1)	27(39.7)	12(44.4)	0.672
**CA-125**				
≤38 U/ml	39(41.1)	34(50.0)	12(44.4)	
>38 U/ml	56(58.9)	34(50.0)	15(55.6)	0.625
**CEA**				
≤5 ng/ml	48(50.5)	36(52.9)	12(44.4)	
>5 ng/ml	47(49.5)	32(47.1)	15(55.6)	0.455
**Therapy**				
Supportive care	12(12.6)	6(8.8)	6(22.2)	
Gemcitabine-based	51(53.7)	39(57.4)	12(44.4)	
5-FU	32(33.7)	23(33.8)	9(33.3)	0.187

Values refer to absolute number (n.) of patients and the corresponding percentage except for interval data. BMI: body mass index, KPS: Karnofsky performance status, CA 19-9: carbohydrate antigen 19-9, CEA: carcinoembryonic antigen, CA-125: carbohydrate antigen-125, NLR: neutrophil-to-lymphocyte ratio.

**Table 2:** Univariate and multivariate Cox-regression analysis for time to treatment failure and overall survival

Factor(n = number of patients with data available)	n. (%)	Univariate analysis		Multivariate analysis	
		TTF	OS	TTF	OS
		HR (95% CI), *p*	HR (95% CI), *p*	HR (95% CI), *p*	HR (95% CI), *p*
**Age (n=95)**					
≤65 years	66(69.5)	1	1		
>65 years	29(30.5)	1.359(0.813-2.272), 0.240	0.934(0.500-1.742), 0.829		
**Gender (n=95)**					
Male	56(58.9)	1	1		
Female	39(41.1)	0.754(0.465-1.223), 0.251	0.666(0.357-1.249), 0.203		
**BMI (n=95)**					
18.5-24 kg/m^2^	66(69.5)	1	1		
≤18.5 kg/m^2^	17(17.9)	1.289(0.683-2.433),	1.958(0.944-4.062)		
>24 kg/m^2^	12(12.6)	0.672(0.327-1.382), 0.351	0.694(0.268-1.799), 0.100		
**KPS (n=95)**					
≤80	40(42.1)	1	1		
>80	55(57.9)	0.937(0.584-1.502), 0.787	0.837(0.461-1.520), 0.558		
**Smoking (n=95)**					
No	64(67.4)	1	1		
Yes	31(32.6)	1.330(0.815-2.171), 0.254	1.188(0.632-2.231), 0.593		
**Alcohol intake (n=95)**					
No	79(83.2)	1	1		
Yes	16(16.8)	1.462(0.811-2.638), 0.207	1.564(0.767-3.186), 0.215		
**Family history of Cancer (n=95)**					
No	64(67.4)	1	1		
Yes	31(32.6)	1.028(0.537-1.968), 0.934	0.694(0.273-1.762), 0.440		
**Diabetes at diagnosis (n=95)**					
No	40(42.1)	1	1		
Yes	55(57.9)	1.068(0.649-1.759), 0.796	1.022(0.549-1.904), 0.945		
**Tumor Location (n=95)**					
Head	40(42.1)	1	1		
Others	55(57.9)	0.860(0.533-1.389), 0.538	0.834(0.458-1.518), 0.551		
**T Stage (n=95)**					
T1, T2	42(44.2)	1	1		
T3, T4	53(55.8)	1.430(0.891-2.296), 0.137	1.215(0.670-2.202), 0.522		
**N stage (n=95)**					
N0	25(26.3)	1	1	1	1
N1	70(73.7)	2.271(1.276-4.043), 0.005	3.225(1.419-7.326), 0.005	1.422(0.757-2.671), 0.273	3.180(1.288-7.850), 0.012
**M Stage (n=95)**					
M0	26(27.4)	1	1	1	1
M1	69(72.6)	3.715(1.981-6.968), 0.0001	5.359(2.067-13.893), 0.001	2.148(0.997-4.626), 0.051	1.306(0.410-4.160), 0.651
**Histology (n=95)**					
PDA	83(87.4)	1	1		
Others	12(12.6)	1.417(0.741-2.710), 0.292	1.452(0.643-3.281), 0.369		
**CA 19-9 (n=95)**					
≤1000 U/ml	56(58.9)	1	1	1	1
>1000 U/ml	39(41.1)	1.868(1.142-3.054), 0.013	2.987(1.608-5.550), 0.001	1.434(0.815-2.526), 0.212	1.981(0.953-4.117), 0.067
**CA-125 (n=95)**					
≤38 U/ml	46(48.4)	1	1	1	1
>38 U/ml	49(51.6)	1.999(1.230-3.249), 0.005	2.209(1.176-4.150), 0.014	1.393(0.832-2.333), 0.207	1.382(0.710-2.688), 0.341
**CEA (n=95)**					
≤5 ng/ml	48(50.5)	1	1	1	1
>5 ng/ml	47(49.5)	1.714(1.062-2.765), 0.027	3.112(1.639-5.906), 0.001	1.021(0.583-1.786), 0.942	1.716(0.790-3.726), 0.173
**Pretreatment NLR (n=95)**					
≤4.0	68(71.6)	1	1	1	1
>4.0	27(28.4)	4.067(2.362-7.002), 0.0001	4.572(2.434-8.589), 0.0001	3.158(1.805-5.527), 0.0001	4.090(2.073-8.071), 0.0001
**Pretreatment PLR (n=95)**					
≤169	56(58.9)	1	1		
>169	39(41.1)	1.511(0.940-2.431), 0.088	1.683(0.917-3.089), 0.089		
**Type of therapy (n=83)**					
Gemcitabine-based	51(61.4)	1	1		
5-FU	32(38.5)	1.141(0.683-1.908), 0.614	1.525(0.776-2.995), 0.217		

Only significant results (*p*<0.05) in the univariate analysis are run in the multivariate analysis. CI: confidence interval, HR: hazard ratio, TTF: time to treatment failure, OS: overall survival, BMI: body mass index, KPS: Karnofsky performance status, PDA: pancreatic ductal carcinoma, CA 19-9: carbohydrate antigen 19-9, CA-125: carbohydrate antigen-125, CEA: carcinoembryonic antigen, NLR: neutrophil-to-lymphocyte ratio, PLR: platelet-to-lymphocyte ratio, 5-FU:5-fluorouracil.
